# Women hormones and hypersensitivity: allergic diseases in menopause

**DOI:** 10.3389/falgy.2026.1777688

**Published:** 2026-04-08

**Authors:** Elitsa Valerieva, Mariela Vasileva, Krasimira Baynova, Borislava Krusheva, Elena Petkova, Miroslava Nenova, Plamena Novakova, Maria Staevska, Stefan Cimbollek, Anna Valerieva

**Affiliations:** 1 Dr. Shterev Hospital, Sofia, Bulgaria; 2 Allergology Department, Spanish National Center for Angioedema, Virgen del Rocío University Hospital, Seville, Spain; 3 Department of Allergology, Medical University of Sofia, University Hospital “Alexandrovska”, Sofia, Bulgaria

**Keywords:** allergic rhinitis, angioedema, asthma, histamine, hormonal changes, hormone replacement therapy, mast-cells, menopause

## Abstract

Menopause is a midlife endocrinological transition that profoundly affects immune regulation, vascular function, and tissue homeostasis, influencing the onset, severity, and clinical expression of allergic diseases. Declining and fluctuating estrogen and progesterone levels modulate mast-cell activity, T2 inflammation, and vascular permeability, contributing to distinct phenotypes in asthma, allergic rhinitis, chronic cough, skin allergies, drug hypersensitivity, anaphylaxis, and angioedema. Clinical observations suggest menopause may exacerbate existing conditions or trigger new-onset disease, with hormone replacement therapy (HRT) potentially modifying disease trajectories. Obesity, comorbidities, polypharmacy, and age-related physiological changes further shape symptom patterns and therapeutic responses. Despite increasing recognition of these effects, mechanistic understanding remains limited, and evidence-based guidelines for diagnosis, management, and individualized therapy in peri- and postmenopausal women are scarce. This review synthesizes current knowledge on hormonal influences in allergic diseases, highlights menopause-specific clinical considerations, and identifies major research gaps. Understanding the interplay between sex hormones, immune function, and allergic disease expression is critical for optimizing care. Clinicians should integrate peri-/menopause status into assessment and management, and future research should aim to clarify pathophysiologic mechanisms, risk factors, and tailored interventions for women in midlife.

## Background

Ageing is a gender-specific phenomenon that in women is strongly influenced by the menopausal transition. This endocrinological continuum leads from regular ovulatory cycles to the final menstrual period associated with ovarian senescence. Menopause is defined as the cessation of menstruation for 12 consecutive months and typically occurs around the age of 50 (1). Perimenopause precedes menopause and is characterized by fluctuating ovarian function, irregular menses, and symptoms such as vasomotor disturbances, sleep disruption, mood changes, and vaginal atrophy, often lasting several years (2). Postmenopause follows menopause, during which symptoms may persist or gradually abate, while health risks related to estrogen deficiency—such as bone loss and cardiovascular changes—become more prominent. Menopause may occur prematurely due to disease or iatrogenic causes; menopause before age 40 is defined as premature, and between 40 and 45 years as early menopause (3).

**Figure 1:**
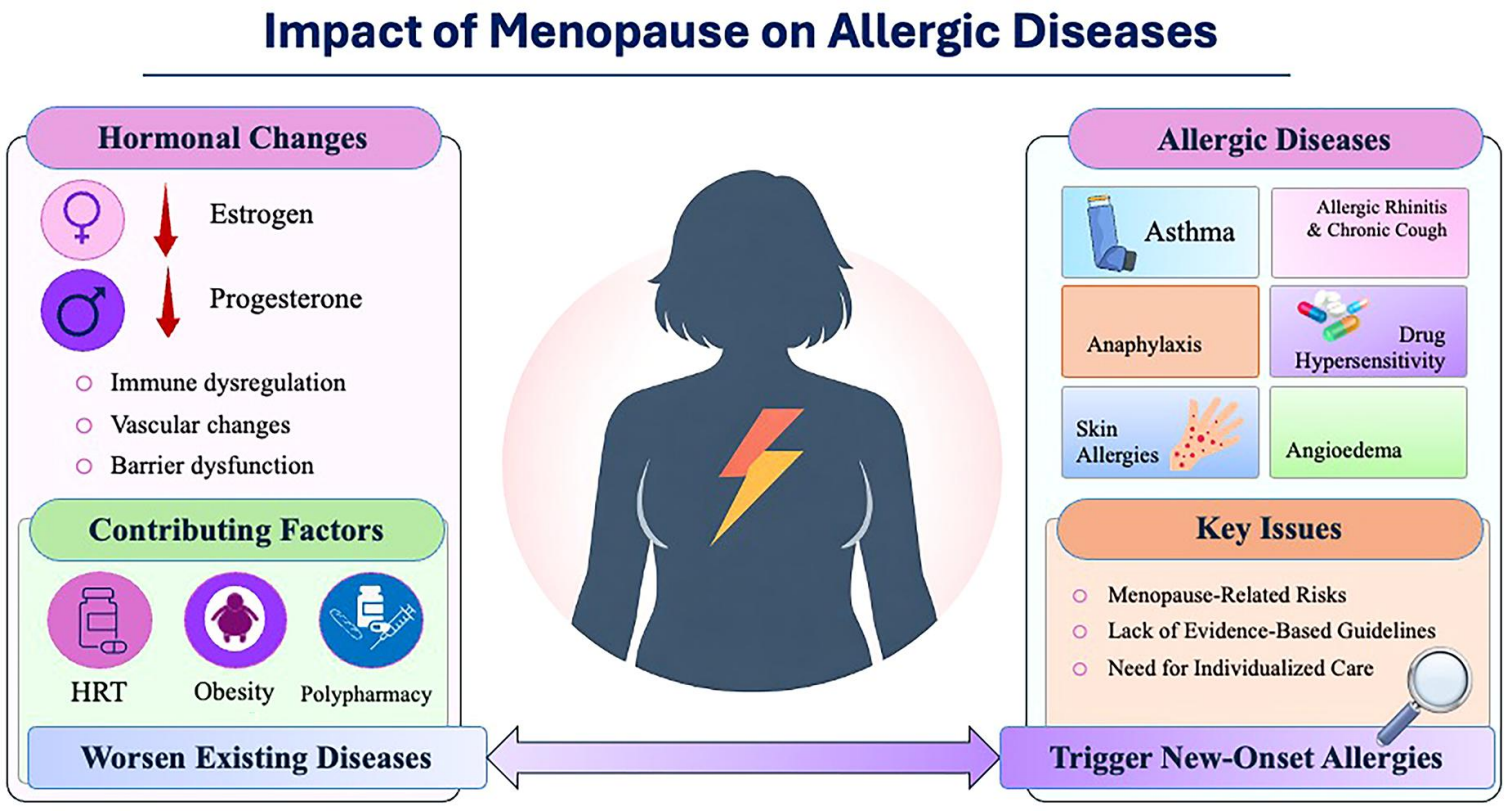
Graphical abstract: impact of menopause on allergic diseases. HRT - hormone replacement therapy.

**Table 1:** Impact of menopause and hormonal changes across allergic and hypersensitivity diseases.

Condition	Menopause-related pattern	Mechanisms (key points)	HRT/exogenous hormone effects	Practical clinical implications
Asthma	Post-puberty prevalence higher in women; postmenopause (esp. surgical) ↑ new-onset risk; BMI partly mediates.	Estrogen/progesterone promote T2 inflammation; ERα → ↑CRTh2/T2; androgens suppress T2 (↓ILC2); possible postmenopausal low-estrogen phenoendotype.	Evidence mixed; estrogen-only often linked to ↑ incidence/severity/exacerbations; may worsen T2-high asthma.	Individualize; consider phenotype, BMI, smoking; monitor closely after HRT initiation; reassess if symptoms worsen/new asthma.
Allergic rhinitis (AR)	Symptoms may shift in menopause; diagnosis overlaps with vasomotor rhinitis/GERD/non-allergic rhinitis.	Hormone fluctuations affect mucosal sensitivity, immune regulation, airway reactivity.	HRT associated with ↑ rhinitis (esp. non-allergic); adiposity may amplify risk.	Standard AR therapy (INS, antihistamines, avoidance, ±AIT); consider HRT as trigger → individualized use/monitoring.
Anaphylaxis	Adult triggers: medications/insect venom > foods; in menopause more CV-dominant presentation and delayed recovery; comorbidity/medications influence severity.	Hypoestrogenism alters mast cells/endothelium/T-cell balance; immune remodeling/low-grade inflammation; changes in mast-cell homeostasis and mediator responses	HRT may reintroduce triggers in sensitized women; monitor during initiation.	Personalized, multidisciplinary; review *β*-blockers/ACEi/NSAIDs; consider mast-cell activation work-up in recurrent cases; VIT effective but individualized.
Skin allergies (general)	Estrogen decline → thinner/drier skin, weaker barrier → ↑ irritant/allergic dermatoses.	↓ collagen/elastin/lipids; ↑ mast-cell reactivity/inflammation; perimenopausal progesterone swings may destabilize.	HRT may improve hydration/elasticity but can modulate inflammation.	Barrier-first care (lipid emollients, gentle cleansing, humidity); hormone-aware assessment; monitor if on HRT.
Atopic dermatitis (AD)	New or worsened eczema; prominent xerosis/pruritus, sleep disturbance.	↓ ceramides/NMF → ↑ TEWL; Th2 + IL-6/TNF-α may rise.	May improve hydration; variable effect on inflammation.	Intensive barrier repair; cautious topical steroids; earlier steroid-sparing options.
Contact dermatitis (CD)	↑ irritant/allergic CD (hands/face/neck) due to impaired barrier recovery.	↓ keratinocyte proliferation/repair → ↑ penetration, prolonged inflammation.	Modest barrier benefit; does not prevent sensitization.	Emollients + gentle cleansing; avoid allergens/irritants; standard CD treatment with stronger barrier focus.
Urticaria	Women predisposed; menopause may ↑ physical triggers (heat/cholinergic).	Estrogen deficiency → ↑ histamine release; ↓ diamine oxidase; autoimmune modulation possible.	Estrogen-dominant HRT may worsen; progesterone-dominant/transdermal may be better tolerated.	Control triggers; adjust/stop HRT if worsening; consider thyroid autoimmunity screening.
Drug hypersensitivity (DH)	Self-reported DH ↑ with age; women >55 at higher risk; polypharmacy common.	Estrogens influence immunity/mast-cells/type IV; menopause alters PK (↓ acid, protein binding, P-gp, CYP; ↓CYP1A2 up to ∼50%) → ↑ levels/intolerance/pseudo-allergy; skin changes ↑ topical reactions.	Adds exposure burden; effects individualized.	Medication review + careful labeling; stop culprit; selected cases: rechallenge/desensitization under supervision.
Multiple Drug Intolerance Syndrome (MDIS)	Intolerance to ≥3 unrelated drugs; ∼2.1%–10% population; more common in women; onset ∼57–68; severe reactions uncommon.	Non-immune/iatrogenic; linked to anxiety/depression, ageing, overweight, multimorbidity.	Not specific; may contribute via polypharmacy.	Avoid over-diagnosing “allergy”; rationalize medications; specialist assessment for de-labeling.
Hereditary Angioedema (HAE)	Variable course: some improve post-natural menopause; others worsen/new onset; estrogen-HRT can unmask/worsen (esp. HAE-nC1INH); ACEi/neprilysin inhibitors ↑ risk; acquired C1INH deficiency can appear midlife.	Estrogens ↑ factor XII/KKS → ↑ bradykinin; hormonal shifts affect permeability and bradykinin metabolism.	Estrogen-containing HRT often problematic; progesterone-only/non-hormonal preferred.	Recurrent angioedema without urticaria → complement/C1INH testing + medications review; hormone-aware multidisciplinary care.

ACEi, angiotensin converting enzyme inhibitor; AD, atopic dermatitis; AE, angioedema; AIT, allergen immunotherapy; AR, allergic rhinitis; BMI, body mass index; C1INH, C1 inhibitor; CD, contact dermatitis; CRTh2, chemoattractant receptor-homologous 2; CV, cardiovascular; DH, drug hypersensitivity; ERα, estrogen receptor α; GERD, gastroesophageal reflux disease; HAE, hereditary angioedema; HAE-nC1INH, hereditary angioedema with normal C1 inhibitor; HRT, hormone replacement therapy; IgE, immunoglobulin E; IL-6, interleukin 6; ILC2, innate lymphoid cells 2; INS, intranasal corticosteroid; KKS, kallikrein kinin system; MDIS, multiple drug intolerance syndrome; NMF, natural moisturizing factor; NSAID, nonsteroidal anti-inflammatory drug; P-gp, P-glycoprotein; PK, pharmacokinetics; T2, Type 2; TEWL, transepidermal water loss; Th2, T helper 2; TNFα, tumor necrosis factor α; VIT, venom immunotherapy.

Humans are unique among terrestrial species in experiencing menopause in a predictable pattern, and its evolutionary significance remains debated (4). With life expectancy now far exceeding reproductive years, menopause is increasingly recognized as a midlife event rather than an endpoint. Women's health is a global priority, particularly in Europe, where women comprise 70% of individuals over 85 years of age—a demographic expected to grow. Despite longer life expectancy, many women experience prolonged ill health, underscoring the need to address menopausal and postmenopausal health across all medical disciplines (5).

Significant gaps persist in menopause research and education. Nearly all preclinical ageing studies ignore menopause, and many clinicians feel inadequately trained, contributing to under-recognition and undertreatment of symptoms (6, 7). Unresolved questions remain regarding menopause-related risks for dementia, ophthalmologic disease, and optimal evidence-based management.

## Women's health in menopause

Menopause-related hormonal changes substantially affect quality of life, general health, and socioeconomic wellbeing (8–10). Major associated health issues include cardiovascular disease, osteoporosis, cancer, metabolic disorders, depression, vasomotor symptoms, sleep disturbances, migraine, and cognitive decline. Estrogen deficiency contributes to adverse body composition changes, increased cardiovascular risk, osteoporosis, and neurocognitive impairment (11–13). Associations between menopause timing and cancer risk remain complex and partly controversial, with inflammation proposed as a contributing mechanism (14, 15).

Hormonal shifts during menopause promote chronic low-grade inflammation and immune dysregulation, leading to reduced immune responses and increased susceptibility to infections in postmenopausal women (Figure 1) (16, 17). Yet, the relationship between menopause and allergic diseases remains poorly investigated. Existing evidence is fragmented and largely extrapolated, leaving major gaps in understanding disease onset, severity, and progression. In this manuscript, we review the current knowledge on this topic, critically appraise available data, and identify unmet needs and future research directions to improve the management of allergic diseases in peri- and postmenopausal women.

## Clinical and management considerations in menopausal women with allergies

### Bronchial asthma

Asthma in women is closely linked to hormonal changes across the reproductive lifespan, including menopause (Table 1). Approximately 15%–22% of women with asthma are menopausal, and menopause affects an estimated 18% of asthmatic women worldwide (18). Asthma prevalence shifts after puberty, resulting in higher rates in women than men, a change attributed to increasing female sex hormones that promote type 2 (T2) inflammation (19, 20).

Longitudinal studies suggest that postmenopausal women, particularly after surgical menopause, have an increased risk of new-onset asthma (21). The impact of hormone replacement therapy (HRT) remains controversial: some studies associate HRT with reduced asthma risk, while others report increased incidence, severity, or exacerbations, especially with estrogen-only regimens (22–24). Recent data indicate that later age at natural menopause may be linked to higher asthma incidence, though these findings require confirmation (25). Importantly, increased asthma risk appears partly mediated by higher body mass index (BMI), as adipose tissue is a key estrogen source in postmenopausal women (26).

Mechanistic insights: Estrogens generally enhance immune responses, while androgens suppress them. Declining estrogen and testosterone levels during menopause alter immune regulation, with estrogens and progesterone promoting T2 inflammation and androgens inhibiting it (27). Estrogen receptor α activation can amplify T2 pathways via CRTh2 upregulation, contributing to asthma severity and steroid insensitivity in some women (20). Conversely, androgens reduce ILC2-driven inflammation. A distinct postmenopausal asthma phenoendotype associated with low estrogen has been proposed, while HRT-related estrogen increases may exacerbate T2-high asthma or increase asthma risk (28).

Clinical implications: Given heterogeneous epidemiologic data but consistent biological mechanisms, management should be individualized. When prescribing HRT, clinicians should consider asthma history, inflammatory phenotype, BMI, smoking status, and patient preferences. Closer monitoring after HRT initiation is advisable in women with asthma. In women without asthma, HRT is not contraindicated, but shared decision-making should address uncertain respiratory effects, with reassessment if new or worsening symptoms occur.

### Rhinitis: allergic, non-allergic and chronic cough

During menopause, allergic rhinitis (AR), non-allergic rhinitis and chronic cough may present with distinct features due to fluctuations in estrogen and progesterone, which can affect immune regulation, mucosal sensitivity, and airway reactivity. Approximately 33% of postmenopausal women report chronic cough lasting longer than eight weeks, even in the absence of pulmonary disease. This symptom burden correlates with higher somato-vegetative and urogenital scores on the Menopause Rating Scale, suggesting increased cough sensitivity during the climacteric period (29). Women also exhibit greater cough reflex sensitivity than men, a difference that may intensify after menopause (30).

Chronic dry cough in menopausal women is often characterized by a non-eosinophilic, CD4+ T-cell–predominant airway inflammation, differing from classic allergic eosinophilic patterns and indicating a shift in inflammatory phenotype (31). In AR, longer lifetime exposure to endogenous estrogens has been linked to higher prevalence later in life (32). HRT has been associated with increased rhinitis symptoms, particularly non-allergic (vasomotor/irritative) rhinitis manifestations such as nasal congestion, rhinorrhea, and hyperreactivity without IgE sensitization, with obesity apearing to worsen the risk for more severe and refractory cases through peripheral estrogen production and pro-inflammatory adipokine signaling (33, 34).

Diagnosis of AR and chronic cough in menopausal women can be challenging due to overlap with vasomotor symptoms, non-allergic rhinitis, and gastroesophageal reflux disease. Allergy testing is essential and management includes avoidance of allergens/irritants, lifestyle measures, and standard therapies such as intranasal corticosteroids and antihistamines, with allergen immunotherapy when clinically relevant. Chronic cough treatment focuses on addressing underlying causes like the post-nasal drip; neuromodulators may be considered in refractory, non-eosinophilic cases. Given the potential for HRT to exacerbate rhinitis, its use should be individualized, balancing symptomatic benefits against possible respiratory effects.

### Anaphylaxis: definition, mechanisms, and hormonal modulation in menopause

Anaphylaxis is a severe systemic hypersensitivity reaction mediated by immune or non-immune pathways that converge on mast-cell and basophil activation, leading to the release of vasoactive and inflammatory mediators and resulting in multi-organ involvement. While food allergens predominate in younger individuals, insect venom and medications are more common triggers in adults. In menopausal women, hormonal changes, vascular factors, and comorbid medication use significantly influence reaction severity and recovery (35, 36).

Beyond metabolic roles, estrogens and progesterone modulate immune and vascular responses. Postmenopausal hypoestrogenism alters mast-cell function, endothelial tone, and T-cell polarization, potentially modifying the clinical presentation and outcomes of anaphylaxis. Estrogens enhance Th2 responses, IgE production, mast-cell degranulation, and increased vascular permeability, whereas progesterone promotes immune tolerance (37). Estrogen loss after menopause may therefore lead to altered hemodynamics, slower onset of anaphylaxis via endothelial nitric oxide signaling, altered vasodilatory capacity, and impaired endothelial barrier regulation, contributing to greater blood pressure variability and cardiovascular instability during systemic reactions. Concurrent immune remodeling characterized by reduced regulatory T-cell activity, low-grade pro-inflammatory myeloid activation involving IL-6 and TNF-α pathways, and context-dependent changes in mast-cell biology, together with chronic low-grade inflammation may explain severe reactions despite lower IgE levels in some postmenopausal women (38).

Food allergy can persist or newly develop after menopause (39). Estrogen deficiency compromises epithelial barriers, mucosal immunity, and gut microbiota, while reduced gastric acidity, motility changes, and frequent use of acid-suppressive drugs facilitate sensitization. Comorbidities and medications common in this age group—such as β-blockers, ACE inhibitors, and NSAIDs—may further exacerbate reaction severity (40). Clinically, menopausal women more often present with cardiovascular-dominant manifestations and delayed recovery compared with premenopausal women (38).

Hymenoptera venom allergy remains a major cause of adult anaphylaxis. In menopausal women, hormonal decline, vascular stiffness, and cardiovascular comorbidities increase the risk of severe reactions compared with younger/premenopausal women. Through both genomic and rapid non-genomic pathways, estrogen signaling modulates mast-cell biology and mediator release; its deficiency may influence mast-cell homeostasis and tissue distribution, potentially contributing to altered mediator responses in some individuals (39). Venom immunotherapy remains highly effective but should be individualized (41).

Hormone-associated hypersensitivity syndromes illustrate the immunomodulatory role of sex hormones (42, 43). Although cyclical anaphylaxis is not reported after menopause, exogenous hormones used in HRT may reintroduce triggers in sensitized women. Careful monitoring during HRT initiation and interdisciplinary collaboration are recommended.

Overall, the interaction between hormonal deficiency, immune remodeling, and vascular changes in menopause modifies anaphylaxis presentation and prognosis (38). Evaluation and management should be personalized, multidisciplinary, and integrated with endocrine and cardiovascular care to optimize outcomes.

### Skin allergies: atopic dermatitis, contact dermatitis, urticaria

Menopause is a major hormonal transition that significantly affects skin structure, barrier integrity, and immune balance. Estrogen supports cutaneous health by stimulating collagen synthesis, enhancing epidermal renewal, regulating sebaceous activity, and maintaining skin hydration (44). With estrogen decline, collagen and elastin production decrease, the skin becomes thinner and drier, lipid synthesis is reduced, and barrier function weakens, increasing susceptibility to irritation and inflammation (45). Estrogen deficiency also disrupts cutaneous immune regulation, enhancing mast-cell degranulation and inflammatory responses, while fluctuating progesterone levels during perimenopause may further destabilize immune balance (46). Together, these changes predispose menopausal women to allergic dermatoses (47).

### Atopic dermatitis (AD)

Menopausal skin is particularly prone to xerosis and inflammation due to impaired barrier repair and reduced lipid synthesis. Declining estrogen lowers ceramide and natural moisturizing factor production, increasing transepidermal water loss and irritant susceptibility (45, 48). Immune shifts, including enhanced Th2 cytokine activity and elevated IL-6 and TNF-α, may further aggravate AD. Clinically, menopausal women may develop new-onset or worsening eczema, commonly affecting hands, trunk, or flexural areas, often with significant pruritus and sleep disturbance.

Management considerations: Treatment of AD in menopausal women focuses primarily in the greater need for intensive barrier repair, more cautious use of topical corticosteroids, earlier reliance on steroid-sparing therapies, and individualized consideration of hormonal influences. HRT may improve skin hydration and elasticity; however, it requires careful monitoring, as hormonal modulation may also affect inflammatory activity and disease severity (45).

### Contact dermatitis (CD)

Both irritant and allergic contact dermatitis become more common during menopause due to epidermal thinning, reduced sebum production, and delayed barrier recovery. Estrogen deficiency impairs keratinocyte proliferation and repair, facilitating allergen penetration and prolonged inflammation (45, 48). Clinically, dermatitis frequently involves hands, face, and neck.

Management considerations: Management in menopausal women places particular emphasis on reinforcing the epidermal barrier through the regular use of lipid-rich emollients and gentle cleansing practices, which are essential to compensate for impaired barrier function. Although HRT may modestly enhance epidermal regeneration and improve skin hydration, it does not prevent allergen sensitization or allergic contact reactions (45).

### Urticaria

Chronic urticaria disproportionately affects women, highlighting hormonal influences (45). During menopause, estrogen deficiency alters mast-cell behavior, increasing histamine release in the skin, while reduced diamine oxidase activity impairs histamine degradation (45, 49). Vasomotor instability and heat sensitivity may trigger physical urticaria, particularly cholinergic types, and hormonal fluctuations may modulate autoimmune mechanisms involved in chronic spontaneous urticaria (45). HRT, especially estrogen-dominant regimens, can exacerbate urticaria in susceptible women, whereas progesterone-dominant or transdermal formulations may be better tolerated (45).

Management considerations: Management requires attention to hormonal triggers. Worsening symptoms during HRT may necessitate dose adjustment or non-hormonal alternatives. Control of vasomotor triggers inducing cutaneous vasodilation—such as sudden temperature changes, physical exertion, emotional stress, alcohol consumption, and menopausal hot flashes—as well as screening for thyroid autoimmunity, are recommended (45).

### Conclusion and clinical implications

Effective prevention and management of skin allergies in menopausal women require a hormone-aware, integrative approach. Early barrier-repair strategies—daily lipid-rich emollients, gentle cleansing, and humidity control—can reduce flare risk (48). Peri-/menopause status should be considered when evaluating new or persistent dermatoses, as estrogen and progesterone fluctuations may amplify inflammation or modify treatment responses (45, 46). While HRT can improve skin hydration, it may also influence immune reactivity, necessitating individualized assessment and close monitoring (45). Integrating dermatologic care with peri-/menopause evaluation offers the most effective strategy to maintain skin health during ageing (47).

### Drug hypersensitivity and menopause

Drug hypersensitivity (DH) is closely linked to sex-specific hormonal influences on immune function, contributing to distinct clinical patterns in women, similar to those observed in autoimmune diseases (50). Estrogens enhance humoral immunity, autoimmunity, influence mast-cell activation, and delayed type IV reactions, whereas androgens, progesterone, and glucocorticoids exert immunosuppressive effects (51). Accordingly, women report drug allergies more frequently than men, a difference attributable to both biological factors and gender-related behaviors such as higher healthcare utilization (51).

Self-reported DH increases with age and is particularly common in women over 55 years, in whom female sex represents an independent risk factor (52). Midlife women frequently develop comorbid autoimmune conditions and are exposed to polypharmacy, including HRT, antidepressants, bisphosphonates, antihypertensives, and analgesic or anti-inflammatory drugs, further increasing the likelihood of drug reactions (53). Although menopause itself does not directly cause DH, perimenopausal and menopausal hormonal changes may heighten drug sensitivity or unmask adverse reactions (54).

Declining and fluctuating estrogen levels influence drug absorption, distribution, metabolism, and elimination. Estrogens reduce gastric acid secretion, alter plasma protein binding, affect P-glycoprotein activity, and modulate hepatic cytochrome P450 enzymes—most notably reducing CYP1A2 activity by up to 50%—thereby increasing circulating drug levels and the risk of side effects, intolerance, or pseudo-allergic reactions (54). As discussed, menopausal skin thinning and dryness increase susceptibility to contact dermatitis from topical medications, transdermal patches, and cosmetic products.

A condition highlighting sex- and age-related vulnerability is multiple drug intolerance syndrome (MDIS), a non-immune, partly iatrogenic disorder associated with anxiety and depression (1, 6). MDIS is defined by non-immunological adverse drug reaction that is often dose-related and causes unintended side effects of intolerance to three or more unrelated drug classes and affects 2.1–10.05% of the general population (55, 56). Although specific menopausal prevalence data are lacking, MDIS is more common in women (6.1% vs. 2.9% in men), with median onset ages overlapping the menopausal period (57–68 years), and is associated with ageing, overweight, and multimorbidity (55–57). Clinical manifestations include predominantly cutaneous symptoms, gastrointestinal complaints, headaches, cough, musculoskeletal pain, fever, dermatitis, and hypertension. Severe reactions are uncommon, but management requires discontinuation of culprit drugs and, in selected cases, cautious re-exposure or desensitization, as its indications have recently been broadened to encompass delayed T-cell-mediated, non-immunological, and non-IgE-mediated reactions and the desensitization precedures varies amongst drugs and patients (56, 58).

### Angioedema in menopause: hereditary and rare forms

Angioedema is a potentially severe condition with certain diagnostic complexity, often underrecognized in the general population with certain specific considerations in menopausal women, particularly hereditary angioedema (HAE) and other rare bradykinin-mediated forms. HAE due to C1 inhibitor (HAE-C1INH) deficiency or dysfunction (types I and II), as well as HAE with normal C1 inhibitor (HAE-nC1INH), is strongly influenced by sex hormones (59). Others, like HAE with *PLG* gene variant did not present any estrogen sensitivity. Estrogens enhance hepatic synthesis of factor XII, activate the kallikrein–kinin system, and increase bradykinin generation, thereby lowering the threshold for angioedema attacks. Hormonal fluctuations, particularly the decline in estrogen and progesterone, modulate vascular permeability, potentially influencing both the frequency and severity of angioedema episodes: estrogens normally modulate components of the renin–angiotensin–aldosterone system (RAAS) and enzymes involved in bradykinin degradation, including angiotensin-converting enzyme (ACE) and aminopeptidase P. Declining estrogen levels may therefore alter the balance between bradykinin production and degradation, contributing to changes in vascular permeability and endothelial reactivity. Progesterone has partially counter-regulatory effects on vascular tone and inflammatory signaling; its decline may further modify endothelial responses to bradykinin (60, 61).

The menopausal transition is associated with heterogeneous clinical trajectories. Some women experience partial attenuation of attack frequency after natural menopause, whereas others develop new-onset angioedema or worsening disease, particularly when exposed to estrogen-containing HRT. Estrogen HRT may unmask latent disease, especially in HAE-nC1INH. Progesterone-only or non-hormonal menopausal therapies are generally better tolerated and should be preferred in patients with HAE (62–64).

Polypharmacy—common in this age group—further increases angioedema risk, particularly with ACE inhibitors and neprilysin inhibitors. Acquired angioedema due to C1 inhibitor deficiency, often associated with autoimmune or lymphoproliferative disorders, may present in midlife or postmenopause (65).

Clinical implications: Recurrent angioedema without urticaria in menopausal women should prompt evaluation for bradykinin-mediated mechanisms, including complement testing and medication review. Multidisciplinary, hormone-aware management is essential.

## Research gaps

Significant knowledge gaps exist regarding allergic diseases in menopausal women. Current studies are largely anecdotal or derived from small cohorts, and there is a lack of prospective, longitudinal research evaluating the impact of menopausal status, HRT, and comorbidities on disease onset and progression. Mechanistic insights into hormonal modulation of mast cells, bradykinin pathways, and vascular permeability in this population remain insufficient. Moreover, evidence-based guidelines for diagnosis, management, and individualized therapy in peri- and postmenopausal women are scarce. Addressing these gaps is critical to improve clinical care and patient outcomes.

## Conclusion and key insights

Menopause introduces hormonal and physiological changes that can influence the course of allergic diseases. Estrogen deficiency, immune remodeling, and vascular alterations may modify disease expression and response to therapy. While observational evidence suggests a potential link between menopause, HRT, and allergic diseases, definitive mechanistic and epidemiologic data are lacking. Future research should focus on elucidating hormonal contributions to disease pathomechanisms, identifying risk factors specific to menopausal women, and developing individualized management strategies. Clinicians should remain vigilant for allergic diseases in peri- and postmenopausal women and consider hormonal status as part of comprehensive patient assessment.
